# Transient inhibition of MEK/ERK and WNT pathways enhances direct differentiation of primed hPSCs into functional trophoblast stem cells

**DOI:** 10.1186/s13619-025-00261-x

**Published:** 2026-01-20

**Authors:** Qifan Jiang, Ping Liu, Chunlin Chen

**Affiliations:** Department of Obstetrics and Gynecology, Nanfang Hospital, Southern Medical University, No. 1838, Guangzhou Avenue North, Guangzhou, Guangdong 510515 China

**Keywords:** Human pluripotent stem cells, Human trophoblast stem cells, Placenta, Differentiation, Signaling

## Abstract

The placenta plays a pivotal role in human pregnancy, yet research into placental development has been hindered by limited access to early-stage embryos and ethical constraints. Although human trophoblast stem cells (hTSCs) have been established from blastocysts, deriving these cells efficiently from primed human pluripotent stem cells (hPSCs) remains challenging. Here, we developed a simplified and efficient strategy that enables direct, efficient conversion of primed hPSCs into stable, self-renewing hTSCs by transiently inhibiting the MEK/ERK signaling pathway using the inhibitor PD0325901 in a simplified basal medium. This approach significantly enhanced the generation of trophoblast cells expressing the critical trophoblast marker GATA3 and led to the establishment of homogeneous hTSC lines with robust capacities to differentiate into functional extravillous trophoblast (EVT) and syncytiotrophoblast (STB) lineages. Transcriptomic and chromatin accessibility analyses confirmed that these hTSCs closely resembled blastocyst-derived trophoblast cells and clearly differed from amnion lineages, confirming authentic trophoblast identity distinct from amnion. Additionally, precise modulation of WNT signaling activity was essential for optimal trophoblast induction efficiency, highlighting the importance of signaling equilibrium in trophoblast differentiation. Collectively, our optimized protocol offers an accessible and reproducible platform for modeling early placental development and understanding the pathogenesis of trophoblast-associated disorders in vitro.

**Supplementary Information:**

The online version contains supplementary material available at 10.1186/s13619-025-00261-x.

## Background

The placenta is a vital organ that facilitates maternal–fetal interactions in mammals, playing a crucial role in ensuring normal pregnancy progression by transporting nutrients, exchanging oxygen, secreting hormones, and providing immune protection (Lewis et al. 2012; Turco and Moffett 2019). Among the key cellular components of the placenta, trophoblast cells are of particular significance. These cells originate from the trophectoderm (TE) of the blastocyst, the outermost layer of the pre-implantation embryo, and serve as progenitors of all trophoblast lineages within the placenta (James et al. 2012). Abnormal development of trophoblast cells is closely associated with a range of pregnancy complications, including miscarriage, preeclampsia, fetal growth restriction, and placenta accreta, highlighting the importance of understanding their developmental mechanisms (Burton and Jauniaux 2017; Romero et al. 2011). Despite the importance of trophoblast cells in pregnancy, research on their development and function has been hindered by limited access to early human embryos and ethical restrictions, preventing a comprehensive understanding of placental development and associated pathologies.

To overcome these limitations, stem cell models have become indispensable for investigating early human developmental processes. In 2018, Okae et al. successfully established human trophoblast stem cells (hTSCs) from early placental tissue and blastocysts using a specialized culture system containing TGF-β pathway inhibitors and WNT pathway activators (Okae et al. 2018). This breakthrough enabled researchers to study trophoblast biology without reliance on embryonic material. Building on this, several groups have developed in vitro culture conditions to derive self-renewing hTSCs, thereby facilitating detailed studies of trophoblast development (Dong et al. 2020; Dong and Theunissen 2022; Guo et al. 2021; Io et al. 2021). Notably, naïve human primed pluripotent stem cells (hPSCs), which represent an earlier developmental state, can be efficiently converted into hTSCs using the SAVECY medium (containing SB431542, A83-01, valproic acid, epidermal growth factor, CHIR99021, and Y-27632), with molecular and transcriptomic analysis confirming their equivalence to blastocyst-derived hTSCs (Karvas et al. 2022). In contrast, primed hPSCs, which are more developmentally advanced, require reversion to a naïve state using transgene-free culture systems such as 5i/L/A or t2i/L/Gö before they can be differentiated into hTSCs. However, this reversion process is time-consuming and costly, prompting researchers to explore whether primed hPSCs can be directly converted into hTSCs without transitioning through the naive state (Gafni et al. 2013; Liu et al. 2017).

The feasibility of directly deriving hTSCs from primed hPSCs remains controversial. Primed hPSCs exhibit transcriptomic similarities to the late post-implantation epiblast, a developmental stage at which the TE and epiblast (EPI) lineages have already diverged (Liu et al. 2018). Additionally, primed hPSCs display significantly higher DNA methylation levels than naive hPSCs, raising concerns about epigenetic barriers to direct trophoblast differentiation (Smith et al. 2014). Previous studies have shown that exposing primed hPSCs to TSC-inducing conditions results in differentiation toward neural ectodermal rather than trophoblast lineages (Dong et al. 2020). Nevertheless, our group previously observed that applying the SAVECY medium to primed hPSCs allowed the emergence of small hTSC-like clones, which could be expanded into stable hTSC lines through clone selection (Wei et al. 2021). However, the efficiency of this approach was extremely low, highlighting the need for optimized methods to enhance TSC induction.

Given these challenges, the present study aimed to establish a more efficient method for deriving hTSCs from primed hPSCs by investigating the role of MEK/ERK pathway inhibition in trophoblast induction. The MEK/ERK pathway is known to influence lineage specification and self-renewal in various developmental contexts (Deathridge et al. 2019; Yu et al. 2018), but its role in overcoming the epigenetic constraints of primed hPSCs for trophoblast conversion remains unclear. Additionally, while WNT signaling plays a critical role in trophoblast differentiation, the precise balance of WNT pathway activity necessary for optimal hTSC induction has not been well defined. To address these gaps, we systematically examined the effects of MEK/ERK inhibition and WNT pathway modulation on hTSC derivation efficiency. By refining the culture conditions for trophoblast induction, this study provides a simplified and robust strategy for generating hTSCs from primed hPSCs, offering a valuable in vitro model for investigating trophoblast development and pregnancy-related disorders.

## Results

### Transient inhibition of MEK/ERK signaling enhances trophoblast induction from primed hPSCs

**Fig. 1:**
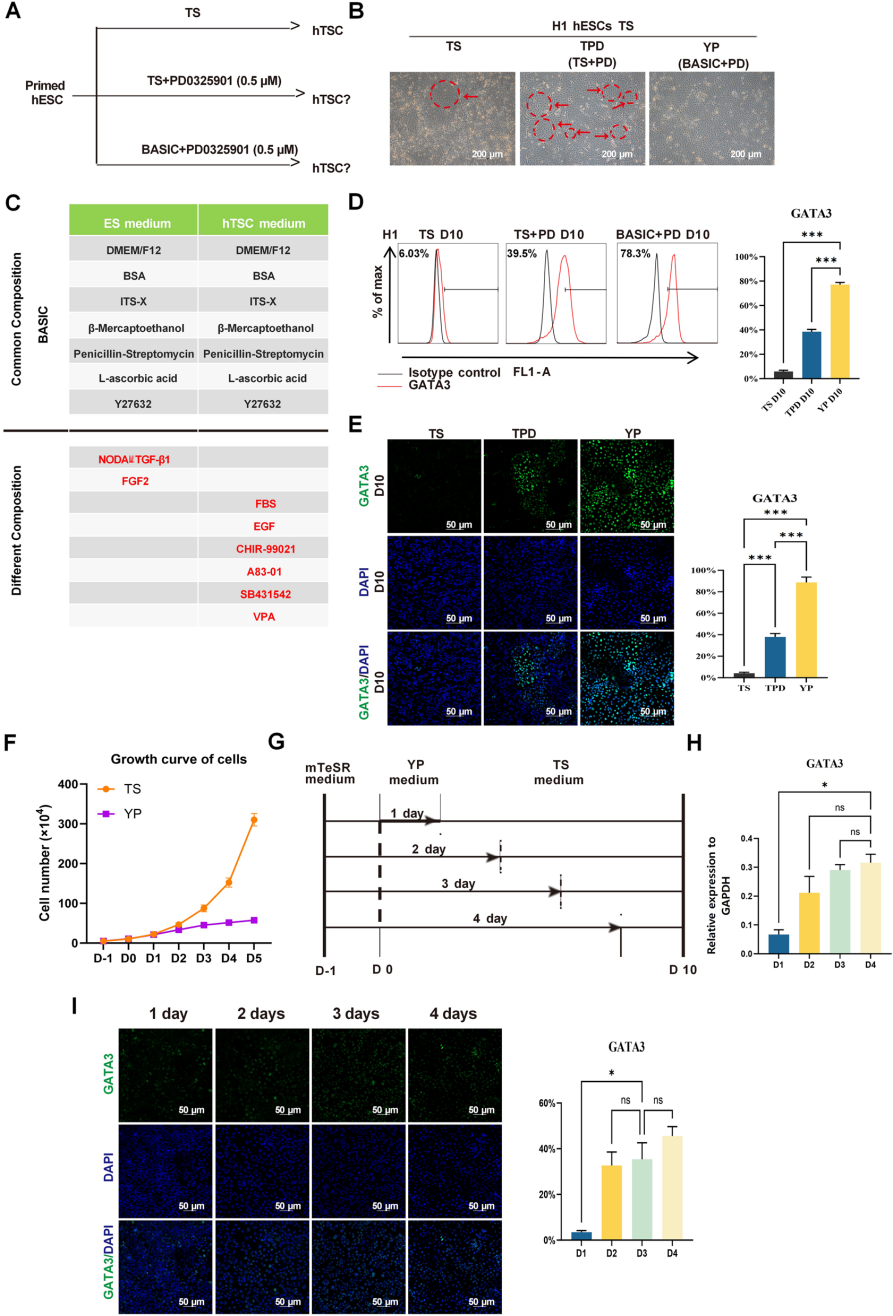
Transient MEK/ERK inhibition enhances trophoblast induction from primed hPSCs. **A**. Schematic of the induction strategies using TS, TS + PD0325901 (TPD), and a simplified YP (Y-27632 + PD0325901) medium to derive hTSCs from primed hPSCs. **B**. Representative morphology of H1 hESC-derived cells cultured under TS, TPD, and YP conditions at Day 10. Scale bar: 200 μm. The arrows indicated hTSC-like clones. **C**. Comparative overview of the major signaling components in hESC and hTSC media formulations. **D**. Flow cytometry analysis of GATA3⁺ cell populations in TS-, TPD-, and YP-treated cultures at Day 10. **E**. Immunofluorescence staining for GATA3 in hTSCs on Day 10 under TS, TPD, and YP conditions. Scale bar: 50 μm. Error bars represent mean ± SD from three biological replicates. **F**. Growth curves of cells under TS and YP conditions, showing proliferation rates. Error bars indicate mean ± SD from three replicates. **G**. Schematic of time-course experiments with variable YP exposure (1–4 days), followed by culture in TS medium. **H**. RT-qPCR analysis of GATA3 expression after different durations of YP treatment. ns, non-significant. *P Error bars represent mean ± SD (*n* = 3). **I**. Immunostaining for GATA3 at different YP exposure durations. Optimal expression and morphology were observed at 1-4 days. Scale bar: 50 μm

We first investigated the role of MEK/ERK signaling inhibition in improving the efficiency of TSC derivation from hPSCs. Cells were treated with the selective MEK/ERK inhibitor PD0325901 (TPD medium) in combination with SAVECY medium, a known TSC-supporting formulation (Fig. 1A). Compared to the TS group, cells in the TPD medium showed increased emergence and expansion of hTSC-like colonies (Fig. 1B). Analysis of medium composition indicated that the fundamental difference between hESC and SAVECY media was primarily related to fibroblast growth factor (FGF) signaling (Fig. 1C). Flow cytometry analysis revealed a significant increase in GATA3-positive cells upon MEK/ERK inhibition (39.5% in TPD at day 10 versus 6.03% in the TS group) (Fig. 1D). Notably, using a simplified basal medium supplemented only with Y-27632 and PD0325901 (YP medium) yielded an even higher proportion of GATA3-positive cells (78.3%) (Fig. 1E). However, cells in the YP group showed a reduced proliferation rate after Day 3 (Fig. 1F), likely reflecting MEK/ERK inhibition’s known role in cell growth regulation, particularly its downstream effects on FGF, EGF, and platelet-derived growth factor (PDGF) signaling. To identify the optimal treatment window, we induced H1 ESCs with YP culture medium for 1–4 days and then maintained the cells in the TS medium (Fig. 1G). Quantitative PCR analysis showed increased GATA3 expression over time, peaking at Day 4 (Fig. 1H). By observing cell morphological changes and detecting TSCs-related marker genes, we found that the MEK/ERK pathway inhibitor treatment for 3 days was an ideal selection for TSCs differentiation. The shorter induction time led to an apparent increase in GATA3-negative cells, while the longer inhibition time did not significantly increase the rate of GATA3-positive cells, and the proliferation of cells was significantly slowed down (Fig. 1I). When using only 1 day of YP medium, apparent cell mixing was visible. In contrast, the cells that were cultivated for 2 days or more in the YP medium transformed into relatively uniform flat epithelial-like cells (Fig. S1A). These findings demonstrate that transient MEK/ERK inhibition significantly enhances TSC induction from primed hPSCs, with a 3-day window providing the optimal balance between induction efficiency and cell viability.

### Transient inhibition of the MEK/ERK pathway enhances the efficacy of primed hPSC-derived TSC-like cells

**Fig. 2:**
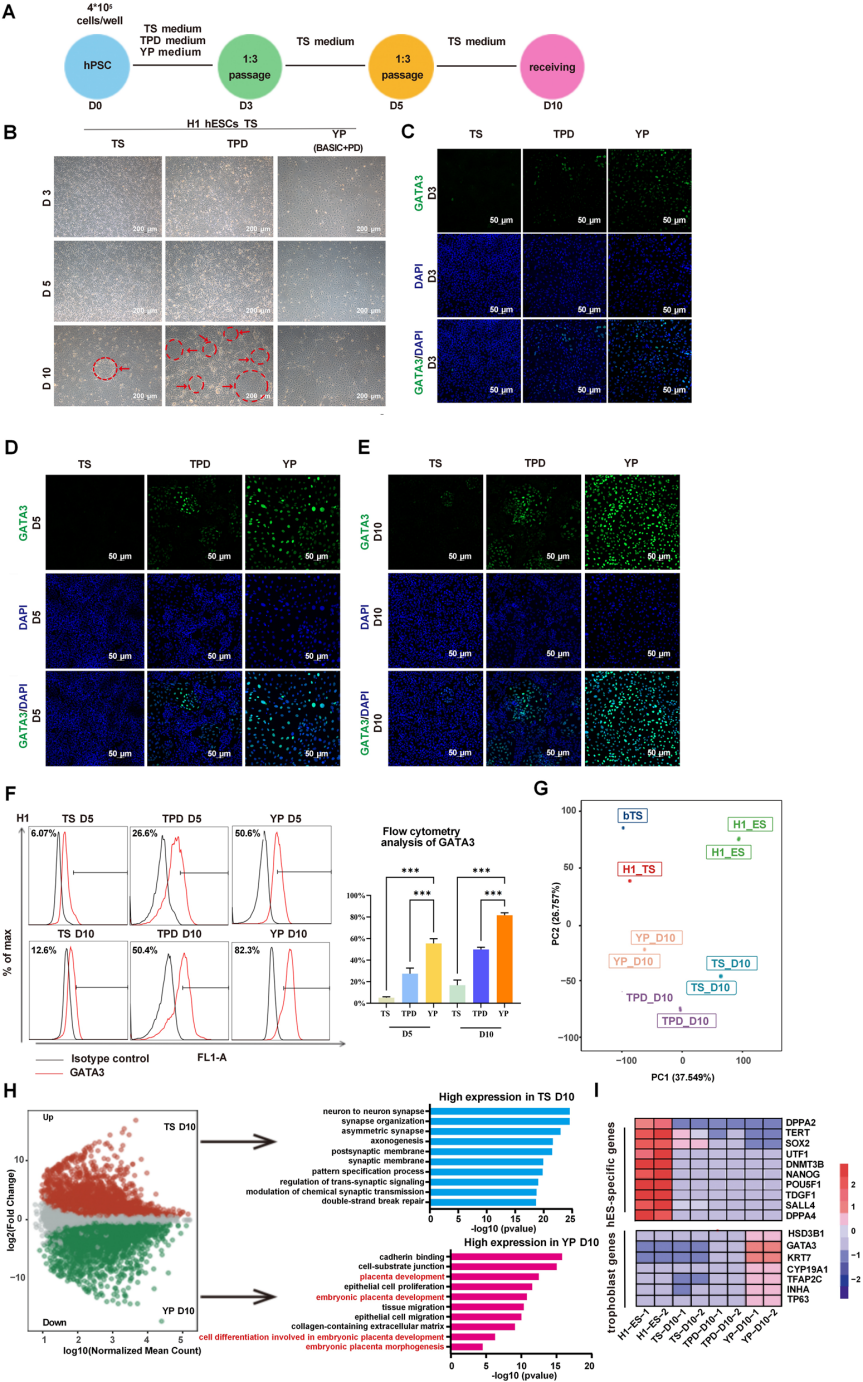
YP treatment efficiently induces hTSCs with trophoblast identity. **A**. Overview of the differentiation strategy comparing TS, TPD, and YP conditions on different days. **B**. Cell morphology on Days 3, 5, and 10 under each condition. YP-treated cells show uniform epithelial morphology (*n* = 5). Scale bar: 200 μm. **C**-**E**. Immunostaining of GATA3 on Days 3, 5, and 10. YP-treated cells show consistently strong GATA3 expression (*n* = 5). The arrows indicated hTSC-like clones. Scale bar: 50 μm. Error bars represent mean ± SD from three independent experiments. **F**. Flow cytometry analysis of GATA3⁺ cells on Day 10 under each condition (*n* = 5). ***P < 0.001. **G**. PCA plot based on RNA-seq of Day 10 cells, comparing them to H1 hESCs, blastocyst-derived TSCs, and other controls. **H**. Left: Volcano plot showing differentially expressed genes between YP and TS Day 10 cells. Right: GO term enrichment for upregulated genes in each condition. **I**. Heatmap of selected trophoblast and pluripotency-associated genes, showing elevated trophoblast markers in YP-derived cells

Having identified 3-day MEK/ERK inhibition (YP condition) as optimal for GATA3 induction, we next compared this strategy to standard TS and TPD conditions to assess the overall efficacy in establishing TSC-like cells (Fig. 2A). The morphological assessment showed well-defined hTSC-like colonies in the TPD condition, while the YP group consistently produced a uniform epithelial morphology characteristic of bona fide hTSCs (Fig. 2B). Immunofluorescence staining confirmed the highest proportion and intensity of GATA3^+^ cells in the YP group over time (Fig. 2C-E). Consistently, flow cytometric analysis indicated earlier and substantially higher GATA3^+^ induction efficiency in the YP group (82.3% at Day 10) compared to the TS and TPD groups (Fig. 2F).

To evaluate the transcriptional similarity of induced cells to primary trophoblasts, we performed RNA sequencing on Day 10 samples. Principal component analysis and hierarchical clustering revealed that the YP group closely resembled primary trophoblast cells, whereas the TS group was transcriptionally divergent (Fig. 2G). Additionally, the TS, TPD, and especially YP group exhibited significantly low or absent expression of marker genes for the three germ layers, including PAX6/SOX1 (ectoderm), CD31/MYB1 (mesoderm), and SOX17/FOXA2 (endoderm), suggesting the specific differentiation under YP treatment (Fig. S2D). Gene ontology analysis of upregulated transcripts in the YP group highlighted enrichment for placenta-associated pathways, while the TS group showed neural lineage enrichment (Fig. 2H). Notably, most pluripotency genes were downregulated in all groups, while trophoblast markers such as GATA3, KRT7, TP63, and TFAP2C were most highly expressed in the YP group (Fig. 2I, S1B). Particularly, OCT4 expression remained stably low across all groups (Fig. S1C), indicating that GATA3 induction occurs prior to full exit from pluripotency. Together, these findings confirm that transient MEK/ERK inhibition primes primed hPSCs toward a trophoblast-like fate with molecular features resembling bona fide TSCs.

To further validate the robustness of our optimized protocol, we repeated the induction experiments using the UH10-hiPSC line. Morphologically, cells derived from UH10-hiPSCs exhibited phenotypes and induction efficiencies comparable to those observed with H1-hESCs (Fig. S2A). RT-qPCR and flow cytometry analyses further supported the superior efficacy of the YP medium, confirming that transient MEK/ERK inhibition consistently promotes efficient trophoblast induction across multiple primed pluripotent stem cell lines (Fig. S2B-C). Overall, these results establish the reproducibility and broad applicability of our method for generating TSCs from both hESCs and hiPSCs.

### Balanced WNT signaling is essential for optimal TSC induction

**Fig. 3:**
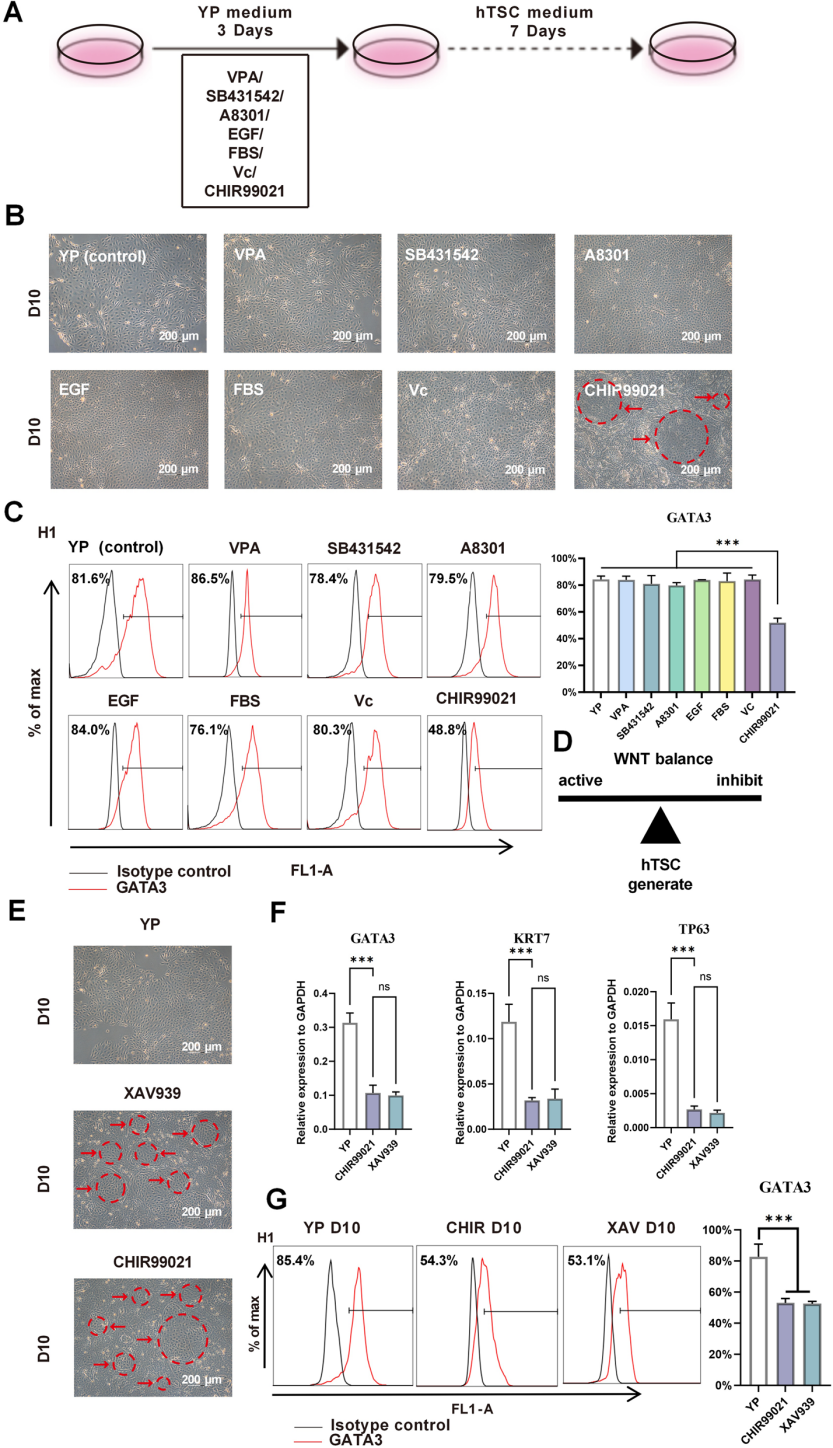
Balanced WNT signaling is essential for optimal TSC induction. **A**. Schematic of the single-factor supplementation strategy: each component from the TS medium was individually added to YP to assess effects on TSC induction. **B**. Representative morphologies of cells treated with individual TS medium components at Day 10. Scale bar: 200 μm. The arrows indicated hTSC-like clones. **C**. Flow cytometry analysis of GATA3⁺ cell populations in response to individual supplements. **D**. Conceptual model: excessive or insufficient WNT signaling disrupts optimal TSC induction. **E**. Morphology of cells treated with YP alone or supplemented with WNT inhibitor (XAV939) or activator (CHIR99021). The arrows indicated hTSC-like clones. Scale bar: 200 μm. **F**. RT-qPCR analysis of TSC marker genes (GATA3, KRT7, TP63) under the same conditions. **G**. Flow cytometry quantification of GATA3⁺ cells across these conditions. ns, non-significant, ***P < 0.001. Error bars denote mean ± SD from three replicates

Given the higher efficacy of the YP medium compared to the TPD medium, we explored whether individual factors in the TS medium negatively affected TSC differentiation. To this end, we added each component of the TS medium individually to the YP medium and monitored differentiation (Fig. 3A). Most additives (e.g., VPA, A83-01, SB431542, EGF, FBS, and vitamin C) did not disrupt the epithelial morphology induced by YP. However, the addition of CHIR99021, a canonical WNT activator, resulted in less uniform morphologies and fewer hTSC-like colonies (Fig. 3B), accompanied by reduced expression of key trophoblast markers (Fig. S1E). Flow cytometry confirmed a significantly lower GATA3^+^ induction rate in the CHIR99021 group (48.8%) relative to the YP-only condition (Fig. 3C). To further dissect the role of WNT signaling, we introduced the WNT pathway inhibitor XAV939 into the differentiation medium (Fig. 3D). As demonstrated by western blot analysis, CHIR99021 treatment significantly upregulated the protein levels of β-catenin and downstream targets such as c-myc and CD44, while exposure to XAV939 showed the opposite inhibition effects on the Wnt/β-catenin signaling (Fig. S3A). Notably, the addition of either CHIR99021 or XAV939 significantly reduced trophoblast marker expression compared to YP control and produced scattered flat cells instead of defined epithelial colonies (Fig. 3E-G). Additionally, the increasing concentration of CHIR99021 or XAV939 treatment gradually decreased the proportion of GATA3^+^ cells (Fig. S3B). Overall, these findings demonstrate that a balanced level of WNT signaling activity is crucial for efficient TSC induction from primed hPSCs, as both excessive activation and inhibition of WNT compromise trophoblast differentiation.

### Established YP-derived TSCs maintain stable TSC features and trophoblast differentiation capacity

**Fig. 4:**
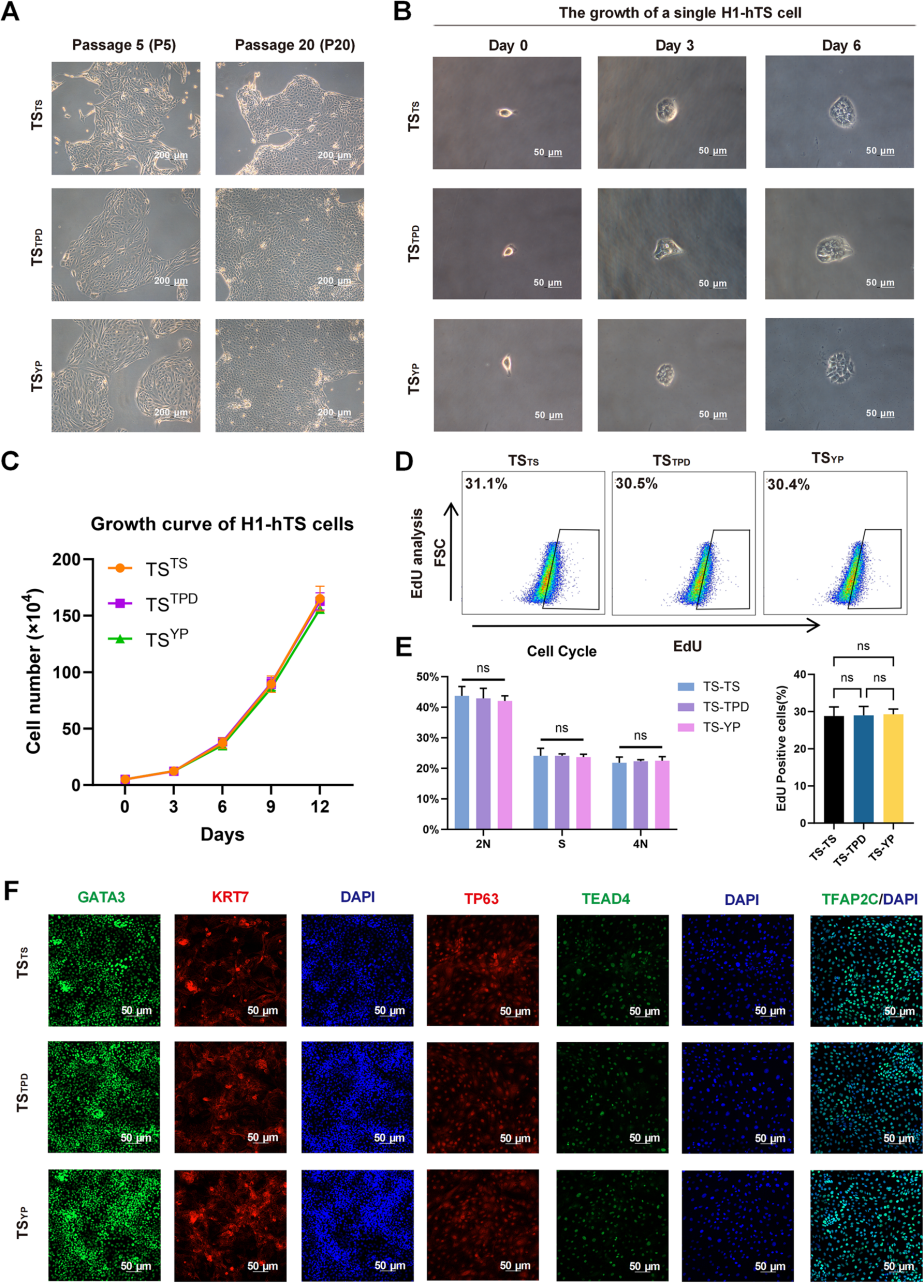
YP-derived hTSCs maintain long-term stability and TSC identity. **A**. Morphology of hTSCs from TS, TPD, and YP groups at early (P5) and late (P20) passages. Scale bar: 200 μm. **B**. Time-lapse phase-contrast images showing the growth of a single hTSC clone. Scale bar: 50 μm. **C**. Growth curves of hTSCs from TS, TPD, and YP groups at passage 20. Error bars show mean ± SD from three replicates. **D**. EdU incorporation assay showing comparable proliferation rates among TSC lines (*n* = 3). **E**. Cell cycle analysis of each hTSC line by flow cytometry (*n* = 3). **F**. Immunofluorescence staining of key TSC markers (GATA3, KRT7, TP63, TEAD4, TFAP2C) in all established lines (*n* = 3). Scale bar: 50 μm. ns, non-significant

To confirm the stability and identity of induced TSC lines, we isolated and expanded hTSC-like colonies generated under TS, TPD, and YP conditions, naming them “TS^TS^”, “TS^TPD^” and “TS^YP^” respectively. All established lines maintained stable epithelial morphology and consistent proliferation characteristics over more than 20 passages (Fig. 4A-C). EdU incorporation and flow cytometry analyses confirmed no significant differences in proliferation and cell cycle profiles among the established TSC lines (Fig. 4D-E). Immunofluorescence analysis demonstrated that all TSC lines uniformly expressed canonical trophoblast markers GATA3, KRT7, TP63, TEAD4, and TFAP2C, closely resembling primary trophoblast stem cells (Fig. 4F). Thus, the induced TSC lines display stable proliferation and authentic trophoblast marker expression, confirming successful TSC establishment from primed hPSCs.

### YP-derived TSCs efficiently differentiate into STB and EVT lineages

**Fig. 5:**
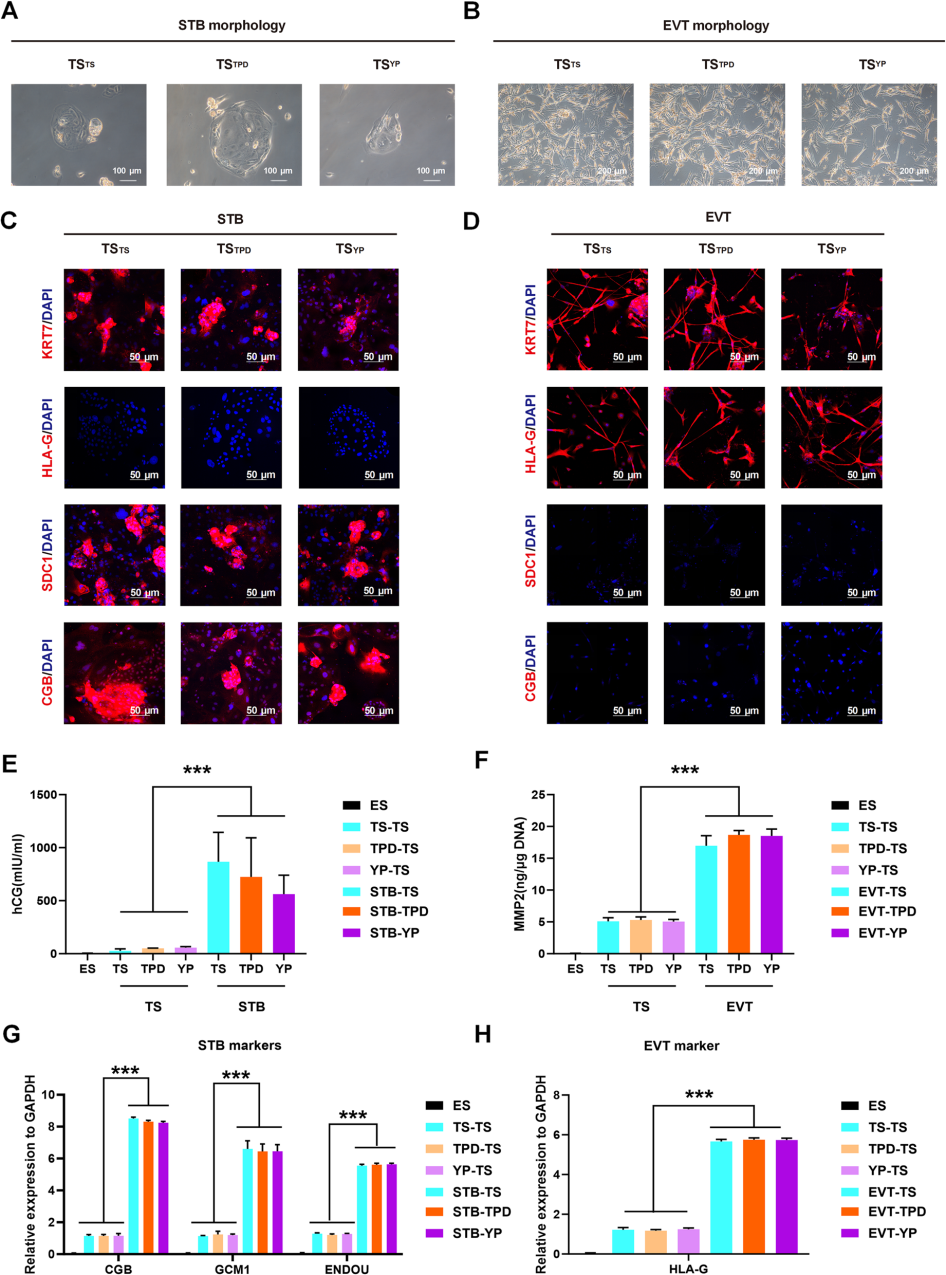
YP-derived TSCs possess the functional capacity to differentiate into STB and EVT lineages. **A**. Morphology of syncytiotrophoblast (STB) cells derived from TSYP upon forskolin stimulation. Scale bar: 100 μm. **B**. Morphology of extravillous trophoblast (EVT) cells induced by NRG1 and Matrigel. Scale bar: 200 μm. **C**. Immunostaining of STB-derived cells for KRT7 (pan-TSC), HLA-G (EVT), and STB-specific markers SDC1 and CGB. Scale bar: 50 μm. **D**. Immunostaining of EVT-differentiated cells confirming HLA-G expression in the absence of STB Markers. Scale bar: 50 μm. **E**. ELISA analysis of hCG secretion in culture media from STB-differentiated cells. Undifferentiated hTSCs and H1-hESCs in mTeSR served as negative controls. **F**. ELISA analysis of MMP2 secretion in culture media from EVT-differentiated cells. **G**. RT-qPCR analysis of expression of STB fusion markers (CGB, GCM1, ENDOU) in each group. **H**. RT-qPCR analysis of expression of EVT marker (HLA-G) in each group. ***P < 0.001. Error bars indicate mean ± SD (*n* = 3)

A defining feature of bona fide TSCs is their differentiation capacity into syncytiotrophoblast (STB) and extravillous trophoblast (EVT). Using established protocols, we evaluated the differentiation potential of the derived TSC lines (Fig. 5A-B). All TSC lines successfully differentiated into STB cells upon stimulation with forskolin, confirmed by morphological fusion and specific expression of STB markers SDC1 and CGB (Fig. 5C). EVT differentiation induced by neuregulin-1 and Matrigel also yielded EVT-like cells expressing EVT-specific marker HLA-G, without STB marker expression (Fig. 5D). Moreover, ELISA detected significant secretion of human chorionic gonadotropin (hCG) from STB-differentiated cells but not undifferentiated TSCs or ESCs, further confirming functional STB identity, and detected the secretion of MMP2 from EVT-differentiated cells rather than undifferentiated TSCs or ESCs (Fig. 5E-F). Additionally, we found that the mRNA expression of markers of STB fusion (CGB, GCM1, ENDOU) showed significant upregulation in STB-differentiated cells compared to the undifferentiated TSCs or ESCs (Fig. 5G), and the expression of EVT marker HLA-G was also demonstrated to be significantly upregulated in EVT-differentiated cells relative to undifferentiated TSCs or ESCs (Fig. 5H). These results collectively demonstrated that YP-derived TSC lines possessed robust and functional differentiation capabilities into EVT and STB trophoblast lineages.

### YP-derived TSCs transcriptome and chromatin accessibility profiles match blastocyst-derived hTSCs and are distinct from amnion lineage

**Fig. 6:**
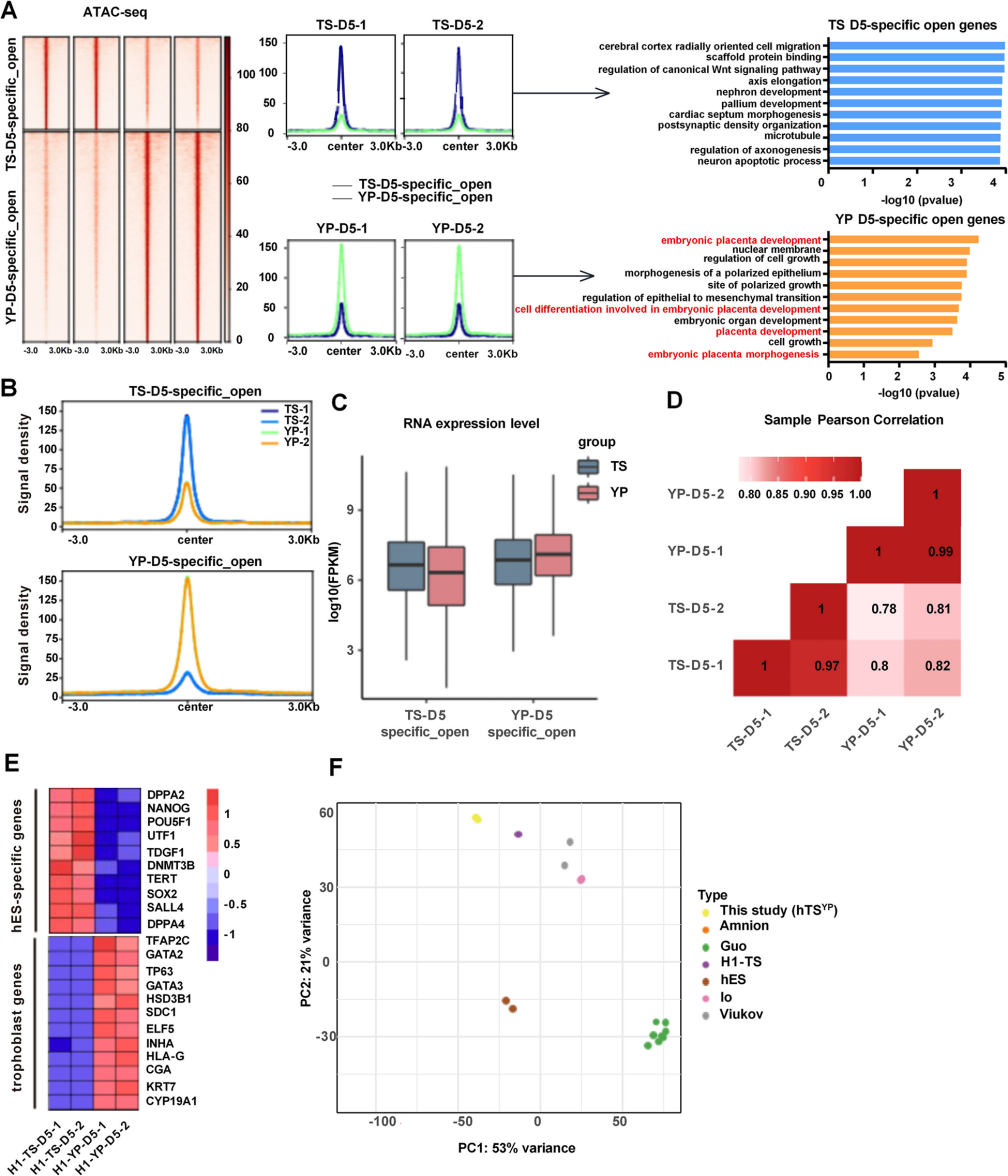
Transcriptomic and epigenomic profiling confirms the trophoblast identity of TSYP cells. **A**. Left: Heatmap of ATAC-seq signal intensity showing differentially accessible chromatin regions between TS Day 5 and YP Day 5 cells. Right: GO enrichment analysis of accessible regions. **B**. Browser tracks showing TS-specific (top) and YP-specific (bottom) ATAC peaks. **C**. RNA expression levels of selected open chromatin (OC) or closed chromatin (CO) genes between groups. **D**. Pearson correlation analysis of whole-transcriptome data between TS D5 and YP D5 cells. **E**. Heatmap showing differential expression of key pluripotency and trophoblast genes across groups. **F**. PCA of TSYP transcriptomes compared to amnion tissues (GSE118808) and previously published TSC models derived from naïve and primed hPSCs (GSE144994, GSE166401, GSE192917)

To further validate the identity of TSYP-derived TSCs, we performed integrated transcriptomic and epigenomic analyses. ATAC-seq revealed significantly increased chromatin accessibility at key trophoblast regulatory loci in the YP-induced cells relative to the TS controls (Fig. 6A-D). RNA-seq confirmed the upregulation of placental genes including TFAP2C, GATA3, HSD3B1, and CYP19A1, alongside silencing of pluripotency factors such as NANOG and SOX2 (Fig. 6E).

These results illustrate that the chromatin accessibility of essential genes for placental development and trophoblast stem cell maintenance is in an open state in hTSCs derived from primed hPSCs. Because amnion and TSCs share some transcriptional overlap (Viukov et al. 2022; Zorzan et al. 2023), we compared TSYP transcriptomes to amnion tissues (GSE118808) and to previously published TSC models derived from naïve hPSCs or blastocysts (GSE144994, GSE166401, GSE192917). Principal component analysis revealed that TSYP cells were distinct from amnion and closely resembled trophoblast models, especially those derived from primed hPSCs (Fig. 6F). Collectively, these data confirm that TSYP cells possess a transcriptional and chromatin identity consistent with genuine trophoblast stem cells and are distinct from both pluripotent and amnion lineages.

## Discussion

In this study, we developed an optimized protocol enabling the direct and efficient derivation of TSCs from primed hPSCs. By transiently inhibiting MEK/ERK signaling using PD0325901 in a simplified basal medium (YP medium), we successfully generated stable, self-renewing TSC lines. These derived TSC lines closely resembled trophoblast cells derived from human preimplantation blastocysts and exhibited robust differentiation capacities into EVT and STB lineages, offering a valuable platform for investigating placental biology and trophoblast-associated diseases.

Previously, the SAVECY medium, a standard medium designed for hTSC culture, demonstrated limited efficacy in directly converting primed hPSCs into stable hTSC lines, typically requiring at least five passages or labor-intensive clone selection to establish stable cultures (Jang et al. 2022; Soncin et al. 2022; Wei et al. 2021). Through comparative analysis of the embryonic stem cell (ES) medium and SAVECY medium, we identified FGF signaling as a critical difference, prompting us to investigate whether modulating FGF-associated pathways could enhance TSC induction efficiency. Although the SAVECY medium already contained TGF-β inhibitors A83-01 and SB431542, which have been reported to promote cytotrophoblast cell proliferation (Okae et al. 2018; Wang et al. 2022), these components alone did not substantially enhance trophoblast induction from primed hPSCs. Therefore, we hypothesized that targeted inhibition of FGF signaling pathways might improve TSC induction efficacy.

FGF signaling, primarily mediated via the MEK/ERK pathway, plays a central role in maintaining pluripotency and regulating the differentiation of human pluripotent stem cells (Dattani et al. 2024; Mossahebi-Mohammadi et al. 2020). By supplementing the basic culture medium with the MEK/ERK inhibitor PD0325901, we created the simplified YP medium. Interestingly, cells cultured in the YP medium consistently adopted a homogeneous, epithelial-like morphology characteristic of bona fide TSCs. This observation aligns with previous findings from Lee et al., who demonstrated that MEK/ERK inhibition facilitated a trophoblast-like cell state by suppressing competing developmental pathways (Lee et al. 2019), and showed significant upregulation of hTSC-related hallmark genes (Balestrini et al. 2024). The mechanisms underlying these observations likely include several possible explanations: (1) MEK/ERK signaling plays a pivotal role in preserving pluripotency and self-renewal potential (Ma et al. 2016); (2) Fibroblast growth factor-2 (FGF2), common in hESC culture, actively drives differentiation towards endodermal and mesodermal lineages (Funa et al. 2008; Shiraki et al. 2008; Yang et al. 2008); and (3) FGF signaling facilitates the differentiation of primitive ectoderm into neuroectoderm, promoting neural lineage commitment (Pollard et al. 2008). Therefore, transient MEK/ERK inhibition effectively blocked unwanted neuroectodermal and mesodermal differentiation pathways, as evidenced by the absent expression of markers for ectoderm, mesoderm and endoderm, thereby promoting trophoblast lineage specification.

Transient inhibition of the MEK/ERK pathway has dual roles: stabilizing primitive pluripotency and simultaneously promoting trophoblast lineage identity (Ma et al. 2016; Smith 2024). Previous research has demonstrated that brief MEK inhibition facilitates the conversion of naïve hPSCs into trophoblast lineage cells, followed by culture in SAVECY medium to generate cytotrophoblast (CTB)-like cells (Dong et al. 2020; Zorzan et al. 2023). Here, our transcriptomic analyses similarly indicated the enrichment of placental developmental genes in the YP-derived cells, contrasting with neural development-related gene enrichment observed under TS medium conditions. Importantly, we also found very low expression of mesodermal markers in YP-derived cells, closely matching blastocyst-derived TSC transcriptomes. Thus, our data suggest that MEK inhibition specifically facilitates trophoblast lineage specification by effectively blocking alternate germ layer differentiation, in agreement with prior findings (Yang et al. 2006).

An ongoing challenge in TSC derivation from hPSCs involves the differentiation into amnion-like cells, due to overlapping transcriptional regulators between trophoblast and amnion lineages. For instance, Guo et al. previously showed that treatment of primed hPSCs with TGF-β and ERK inhibitors resulted in amnion marker upregulation and a global transcriptome indicative of amniotic ectoderm (AME) rather than authentic trophoblast identity, accompanied by low GATA3 expression (Guo et al. 2021). However, our extensive transcriptome analysis and principal component analysis (PCA) clearly distinguished our derived hTSYP lines from amnion-derived cells, clustering them closely with previously validated TSC models reported by Io et al. and Viukov et al. (Oldak et al. 2023). These results suggested that our YP-derived cells represent bona fide trophoblast stem cells rather than amnion derivatives. However, we acknowledge the possibility that a minor fraction of amniotic lineage cells may be present in the induced populations. We regret that the precise proportion of such cells was not quantified, which represents a limitation of our study. Nonetheless, to ensure lineage fidelity, we performed further selection and purification to establish stable TSC lines. Furthermore, compared to previously published protocols for inducing hTSCs from primed hPSCs (Guo et al. 2021; Soncin et al. 2022; Viukov et al. 2022; Wei et al. 2021; Zhang et al. 2025), our study not only underscores the favorable effect of transient MEK/ERK inhibition but also introduces a simplified and efficient induction protocol using YP medium. This method provides novel insights for modeling human placental development using primed hPSCs. Moreover, we confirmed the reproducibility of this approach in both hESC and hiPSC lines, supporting the broad applicability and robustness of our induction strategy.

Furthermore, our observations indicated that the standard TSC maintenance medium (TS medium) negatively affected the induction efficiency compared to the simplified YP medium. To identify the inhibitory components, we tested individual factors of the TS medium separately in combination with the YP medium. We discovered that both activation (CHIR99021) and inhibition (XAV939) of WNT signaling impaired trophoblast induction. These results underscore the importance of balanced WNT/β-catenin signaling in human trophoblast differentiation, and increasing concentration of CHIR99021 or XAV939 decreased the proportion of GATA3^+^ cells in a dose-dependent way, aligning with earlier studies demonstrating that excessive activation or inhibition of the WNT pathway disrupts normal trophoblast development (Chen et al. 2020; Nusse and Clevers 2017). Notably, Huang et al. similarly reported that removing CHIR99021 from the culture medium enhanced the induction of trophoblast fate from primed hPSCs, reinforcing the necessity of maintaining precise WNT signaling homeostasis during trophoblast differentiation (Huang et al. 2014). Mechanistically, this equilibrium likely involves modulation of YAP and TEAD4 transcriptional activity, key determinants in establishing trophoblast identity and lineage commitment (Mizutani et al. 2022). In addition, WNT signaling has been associated with chromatin regions exhibiting open accessibility in hTSCs, suggesting that it may influence the chromatin landscape of differentiation-related genes (Wei et al. 2021). However, the inhibition of WNT signaling has also been linked to differentiation toward EVT lineages, which may interfere with the maintenance of hTSC stemness (Shukla et al. 2024). Interestingly, activation of WNT signaling by CHIR appears to inhibit TSC induction from primed hPSCs, while permitting conversion from naïve hPSCs. Furthermore, the addition of BMP4 to CHIR-containing TS medium has been shown to rescue TSC induction from primed cells, indicating a possible regulatory interplay between WNT and BMP4 pathways in directing cell fate (Viukov et al. 2022; Wei et al. 2021). Together, these findings underscore the importance of maintaining balanced WNT signaling for optimal hTSC derivation from primed hPSCs and highlight the unique advantages of the YP medium in achieving this equilibrium.

In summary, our study establishes a robust, reproducible, and simplified method for generating authentic self-renewing hTSC lines (hTSYP) from primed hPSCs by transient MEK/ERK inhibition. These hTSC lines can readily differentiate into EVT and STB trophoblast subtypes and exhibit transcriptomic profiles resembling genuine human blastocyst-derived TSCs. This optimized protocol provides a valuable in vitro platform for further investigation into placental development and pathologies, facilitating new insights into trophoblast biology. Future research will leverage this approach to better understand critical developmental processes and diseases of the human placenta.

## Materials and methods

### Human pluripotent stem cells (hPSCs) culture

hPSCs, including H1 human embryonic stem cells (hESCs) and UH10 human induced pluripotent stem cells (hiPSCs), were used in this study. All cell lines were authenticated and confirmed to be mycoplasma-free using the MycoAlert Mycoplasma Detection Kit (Lonza) before experimentation. Six-well plates were pre-coated with Matrigel Matrix (Corning, 354277) overnight at 4 °C to ensure uniform coating. Thawed hPSCs were cultured in mTeSR1 medium (STEMCELL Technologies, 85850) and maintained at 37 °C in a humidified incubator with 5% CO₂, with daily media changes to support optimal growth. Cells were passaged every 3 days using Accutase (Innovative Cell Technologies, AT104) to gently dissociate them into single-cell suspensions and replated at a 1:3 to 1:4 split ratio onto fresh Matrigel-coated plates. Cell morphology and confluency were routinely monitored using a phase-contrast microscope (Nikon Eclipse Ts2R), and only low-passage hPSCs (≤ passage 15) were used to prevent genetic or epigenetic drift.

### Induction of human primed pluripotent stem cells into trophoblast stem cells (hTSCs)

Six-well plates were coated with Matrigel Matrix (Corning, 354277) and incubated at 37 °C overnight to ensure uniform coating. H1 human embryonic stem cells (hESCs) were dissociated into single-cell suspensions using Accutase (Innovative Cell Technologies, AT104) and plated at a density of 4 × 10^5^ cells per well in mTeSR1 medium (STEMCELL Technologies, 85850) supplemented with 10 μM Y-27632 (Selleckchem, S1049) to enhance cell survival. This time point was designated as Day −1. After 24 h (Day 0), the culture medium was completely replaced with one of three induction media: TS medium, TPD medium, or YP medium.

For the TS induction group, cells were cultured in trophoblast stem (TS) cell medium, consisting of DMEM/F12 supplemented with 0.1 mM 2-mercaptoethanol (Gibco, 21,985–023), 0.2% fetal bovine serum (FBS, Gibco, 16,000–044), 0.5% penicillin–streptomycin (Gibco, 15,140–122), 0.3% bovine serum albumin (BSA, Sigma, A9418), 1% ITS-X (Gibco, 51,500–056), 1.5 μg/ml L-ascorbic acid (Sigma, A8960), 50 ng/ml epidermal growth factor (EGF, PeproTech, AF-100–15), 2 μM CHIR99021 (Selleckchem, S2924), 0.5 μM A83-01 (Tocris, 2939), 1 μM SB431542 (Selleckchem, S1067), 0.8 mM valproic acid (VPA, Sigma, P4543), and 5 μM Y-27632. For the TPD induction group, cells were cultured in TPD medium, which consisted of TS medium supplemented with 0.5 μM PD0325901 (Selleckchem, S1036), a selective MEK inhibitor. For the YP induction group, cells were cultured in YP medium, which contained BASIC medium (DMEM/F12 supplemented with 0.1 mM 2-mercaptoethanol, 0.5% penicillin–streptomycin, 0.3% BSA, and 1% ITS-X) plus 5 μM Y-27632 and 0.5 μM PD0325901.

Cells were maintained under standard conditions in a humidified incubator at 37 °C with 5% CO₂, and all media were renewed daily. After 3 days (Day 3), the culture medium was replaced with TS medium for all groups, and cells were further cultured in TS medium from Day 4 to Day 10. For routine passaging, cells were dissociated every 3 days using TrypLE Express (Gibco, 12605028). 1 mL of TrypLE Express was added to each well and incubated for 10–15 min, followed by gentle resuspension and subculturing at a 1:3 split ratio onto fresh Matrigel-coated plates in TS medium supplemented with 10 μM Y-27632 for the first 24 h post-passaging. On Day 5 and Day 10, cells were harvested for different biological analysis, including flow cytometry, real-time quantitative PCR, and immunofluorescence staining to assess trophoblast lineage marker expression and differentiation efficiency.

### Human trophoblast stem cells (hTSCs) culture

Clones were subjected to purification and expansion to establish stable hTSCs lines. hTSCs were cultured following previously established protocols (Okae et al. 2018). Briefly, isolated hTSC clones were seeded into Matrigel-coated wells and maintained in 2 mL of TS medium. The medium was refreshed every two days, and cells were passaged at a 1:3 ratio using TrypLE Express when they reached approximately 80–90% confluence. All cultures were maintained at 37 °C in a humidified incubator with 5% CO₂. Throughout the culture period, cell morphology and proliferation were routinely monitored to ensure stable trophoblast stem cell characteristics.

### Differentiation of syncytiotrophoblast (STB) and extravillous trophoblast (EVT) lineages

hTSCs were differentiated into terminal trophoblast cell types, following the protocol established by Okae et al. (Okae et al. 2018) with slight modifications. Cells were cultured until they reached 80% confluence, at which point they were dissociated into single cells using TrypLE Express (Gibco, 12605028). Prior to differentiation, six-well plates were pre-coated with Matrigel (Corning, 354277) overnight at 4 °C to ensure uniform adherence. Cells were plated in their respective differentiation media, with medium changes every other day, and harvested for analysis on Day 6 of differentiation.

#### Syncytiotrophoblast (STB) differentiation

To induce STB differentiation, hTSCs were seeded at a density of 1 × 10^5^ cells per well in 2 mL of STB differentiation medium, consisting of DMEM/F12 (Gibco, 11,330–032) supplemented with 0.1 mM β-mercaptoethanol (Invitrogen, 21,985–023), 0.5% penicillin–streptomycin (HyClone, SV30010), 0.3% bovine serum albumin (BSA, Sigma-Aldrich, A9418), 1% ITS-X (Gibco, 51,500–056), 5 μM Y-27632 (Selleckchem, S1049), 2 μM Forskolin (Selleckchem, S2449), 50 ng/mL epidermal growth factor (EGF, PeproTech, AF-100–15), 1 μM cyclic adenosine monophosphate (cAMP, Sigma-Aldrich, A9501), and 4% KnockOut Serum Replacement (Gibco, 10,828-028). Cells were maintained under standard culture conditions (37 °C, 5% CO₂, humidified incubator), with media replaced every 48 h.

#### Extravillous trophoblast (EVT) differentiation

For EVT differentiation, hTSCs were plated at a density of 4 × 10^5^ cells per well in 2 mL of EVT1 differentiation medium, composed of DMEM/F12 (Gibco, 11330-032) supplemented with 0.1 mM β-mercaptoethanol (Invitrogen, 21985-023), 0.5% penicillin–streptomycin (HyClone, SV30010), 0.3% BSA (Sigma-Aldrich, A9418), 1% ITS-X (Gibco, 51500-056), 20 μM Y-27632 (Selleckchem, S1049), 7.5 μM A83-01 (Selleckchem, S2939), 100 ng/mL neuregulin-1 (NRG1, Cell Signaling Technology, 5218), 1 μM XAV-939 (Selleckchem, S1180), 0.2 μM WNT-C59 (Selleckchem, S7037), 0.5 μM PD0325901 (Selleckchem, S1036), 0.5 μM Thiazovivin (Selleckchem, S1459), 2% Matrigel (Corning, 354277), and 4% KnockOut Serum Replacement (Gibco, 10828-028). After 3 days of culture, the medium was replaced with EVT2 differentiation medium, which contained EVT1 medium but without NRG1, WNT-C59, XAV-939, PD0325901, and Thiazovivin, and with Matrigel at a final concentration of 0.5% to facilitate differentiation.

All cultures were maintained in a humidified incubator at 37 °C with 5% CO₂, and cells were monitored daily for morphological changes indicative of STB and EVT differentiation. Differentiated cells were collected on Day 6 for further analysis, including RT-qPCR, immunofluorescence, and flow cytometry, to assess lineage marker expression and differentiation efficiency.

### Enzyme-linked immunosorbent assay (ELISA)

To assess the secretion of hCG, hTSCs were differentiated into STB cells as described in the STB differentiation protocol. Culture supernatants were collected on Day 6 from STB-differentiated cells and stored at −80 °C until further analysis. As a control group, undifferentiated hTSCs were maintained in TS medium, and supernatants were collected on Day 2. hCG levels in the collected supernatants were quantified using a human hCG ELISA kit (Calbiotech, Cat# ELHCG-001) according to the manufacturer’s instructions. Absorbance was measured using a microplate reader (BioTek Synergy HTX or equivalent), and hCG concentration was determined based on a standard curve. For MMP2 quantification, MMP2 levels were measured using a human MMP2 ELISA Kit (Abcam, UK) following the manufacturer’s instructions. After normalizing the results to total DNA content, DNeasy (Qiagen) was used for quantification.

### Real-time quantitative polymerase chain reaction (RT-qPCR)

Total RNA was extracted from cultured cells using the Total RNA Purification Kit (Magen, Cat# R4111-02), following the manufacturer's protocol. The concentration and purity of the extracted RNA were assessed using a NanoDrop 2000 spectrophotometer (Thermo Fisher Scientific) by measuring absorbance at 260/280 nm. Complementary DNA (cDNA) synthesis was performed using a reverse transcription kit (TOYOBO, Cat# FSQ-301), with 1 μg of total RNA as the input template. Reverse transcription was carried out in a 20 μL reaction volume, following the manufacturer's instructions. Quantitative PCR was conducted using ChamQ SYBR qPCR Master Mix (Vazyme, Cat# Q311-02) on a CFX96 Real-Time PCR Detection System (Bio-Rad, Hercules, CA, USA). The reaction volume for each PCR was 10 μL, consisting of 5 μL of ChamQ SYBR qPCR Master Mix, 0.4 μL of each primer (10 μM), 1 μL of cDNA template, and 3.2 μL of nuclease-free water. Primer sequences used for RT-qPCR are provided in Supplementary Table 1. Gene expression levels were normalized to GAPDH, which served as an endogenous control. Relative gene expression levels were calculated using the 2^−ΔΔCt^ method.

### Immunofluorescence analysis

For immunofluorescence analysis, cells were cultured on glass coverslips placed in 24-well plates and allowed to adhere under standard culture conditions (37 °C, 5% CO₂, humidified incubator). Cells were then fixed with 4% paraformaldehyde (PFA, Sigma-Aldrich, P6148) in phosphate-buffered saline (PBS) for 20 min at room temperature (RT). Following fixation, cells were permeabilized using 0.3% Triton X-100 (Sigma-Aldrich, T8787) in PBS for 10 min at RT. To minimize non-specific antibody binding, cells were blocked with a blocking buffer containing 0.3% Triton X-100 and 10% goat serum (Sigma-Aldrich, G9023) in PBS for 1 h at RT. After blocking, cells were incubated with primary antibodies against anti-TEAD4 (ab58310; Abcam, USA), anti-CGB (ab9582; Abcam, USA), anti-SDC1 (ab34164; Abcam, USA), anti-KRT7 (ZM-0071; ZSGB-BIO), anti-TP63 (13,109; Cell Signaling Technology), anti-HLA-G (NB500-314; Novus Biologicals), anti-Oct-3/4 (sc-5279; Santa Cruz, USA), anti-Gata3 (5852; Cell Signaling Technology), diluted in blocking buffer overnight at 4 °C on a rocking platform to ensure uniform antibody distribution. The following day, cells were washed three times with PBS and incubated with fluorophore-conjugated secondary antibodies for 1 h at RT in the dark to prevent photobleaching. To visualize nuclei, cells were counterstained with 4′,6-diamidino-2-phenylindole (DAPI, Sigma-Aldrich, D9542) diluted 1:5000 in PBS for 5 min at RT. After the final washes, coverslips were mounted using ProLong Gold Antifade Mountant (Thermo Fisher Scientific, P36930) and allowed to cure before imaging. Fluorescent images were acquired using a Laser Scanning Confocal Microscope (LSM 800, Carl Zeiss), and image acquisition was performed using ZEN 2012 software (Carl Zeiss, Jena, Germany). Image analysis, including quantification of fluorescence intensity and co-localization analysis, was conducted using ImageJ software (National Institutes of Health, USA).

### Western blot analysis

Total protein was extracted from TSCs using radioimmunoprecipitation assay lysis buffer. The protein sample was loaded on a 10% sodium dodecyl sulfate–polyacrylamide gel electrophoresis and transferred onto polyvinylidene fluoride membranes (Millipore, USA). Next, the membranes were blocked with 5% skim milk, and incubated with primary antibodies anti-MEK1/2 (1:20,000), anti-p-MEK1/2 (1:5000), anti-ERK1/2 (1:1000), anti-p-ERK1/2 (1:5000), anti-β-catenin (1:5000), anti-c-myc (1:1000), anti-CD44 (1:1000) (Abcam, UK) overnight at 4 °C, with GAPDH (Abcam, UK) as a loading control. Then the membranes were washed and cultured with the secondary antibody (1:2000) for 1 h at room temperature. The enhanced chemiluminescence (ECL) detection kit (Beyotime, PR China) was used for the visualization of the immunobands.

### Flow cytometry analysis

Cells were dissociated into single-cell suspensions using Accutase (Sigma-Aldrich) and incubated at 37 °C for 5–10 min with gentle pipetting to ensure uniform dissociation. Cells were then centrifuged at 300 × g for 5 min, washed once with PBS, and resuspended in 200 μL of 4% paraformaldehyde (BD Biosciences) to fix the cells at room temperature in the dark for 20 min. After fixation, cells were permeabilized by adding pre-cooled 1 × permeabilization solution (BD Biosciences), prepared from 10 × stock solution, and incubated at 4 °C for 10 min. Following permeabilization, cells were resuspended in PBS containing 1% BSA and incubated with primary antibodies for 30 min at 37 °C with gentle agitation. After primary antibody incubation, cells were washed three times with PBS containing 1% BSA and incubated in the dark with fluorescently conjugated secondary antibodies for 30 min at room temperature. After staining, cells were washed again three times with PBS to remove excess secondary antibody and then resuspended in 500 μL of PBS for analysis. Flow cytometry was performed using a BD Accuri C6 Plus Flow Cytometer (BD Biosciences), and at least 10,000 gated events were collected per sample. Data were analyzed using FlowJo software (FlowJo, BD Biosciences), with fluorescence compensation and gating strategies optimized based on fluorescence-minus-one (FMO) controls and unstained controls.

### Apoptosis and EdU proliferation analysis

#### Apoptosis assay

Apoptosis analysis was performed using the FITC Annexin V/PI Apoptosis Detection Kit (KeyGEN, KGA108) according to the manufacturer’s protocol. Cells were dissociated into single-cell suspensions using Accutase (Sigma-Aldrich, A6964) and collected by centrifugation at 300 × g for 5 min at 4 °C. The supernatant was discarded, and cells were resuspended in 70% ethanol and fixed overnight at 4 °C to allow permeabilization for subsequent staining. After fixation, cells were centrifuged at 300 × g for 5 min, the ethanol was removed, and cells were washed twice with PBS. 100 µL of RNase A (100 µg/mL, Sigma-Aldrich, R6513) was added to each sample and incubated at 37 °C for 30 min to degrade residual RNA that could interfere with propidium iodide (PI) staining. Following RNase treatment, 400 µL of PI (50 µg/mL, Sigma-Aldrich, P4170) was added to each sample and incubated at 4 °C in the dark for 30 min to stain DNA. After staining, cells were resuspended in 500 µL of Annexin V Binding Buffer and immediately analyzed using a BD Accuri C6 Plus Flow Cytometer (BD Biosciences). A minimum of 10,000 events were collected per sample, and apoptotic populations (Annexin V-positive, PI-negative for early apoptosis; Annexin V-positive, PI-positive for late apoptosis) were quantified using FlowJo software (BD Biosciences). Unstained and fluorescence-minus-one (FMO) controls were used to establish proper gating parameters.

#### EdU proliferation assay

Cell proliferation was assessed using the Click-iT Plus EdU Pacific Blue Flow Cytometry Assay Kit (Thermo Fisher Scientific) following the manufacturer’s protocol. Briefly, TSCs cells were seeded in differentiation medium containing 10 μM 5-ethynyl-2′-deoxyuridine (EdU) for 3 h. A negative control group was cultured in medium without EdU under identical conditions. After EdU incorporation, cells were dissociated into single cells using Accutase, collected by centrifugation at 300 × g for 5 min, and fixed in Fixation/Permeabilization buffer (BD Biosciences) for 20 min at room temperature. Cells were then permeabilized using Permeabilization Buffer (BD Biosciences) at 4 °C for 10–15 min to allow intracellular staining. For EdU detection, cells were incubated in a reaction buffer containing CuSO₄ and Alexa Fluor 647 azide for 1 h at room temperature in the dark to facilitate click chemistry-based fluorescent labeling of incorporated EdU. Finally, samples were washed, resuspended in PBS, and analyzed using a BD Accuri C6 Plus flow cytometer (BD Biosciences). Data were processed using FlowJo software, and appropriate gating strategies were applied to exclude debris and doublets.

### RNA sequencing and data analysis

Total RNA was extracted from two independent biological replicates using the TRIzol reagent (Invitrogen) following the manufacturer's protocol. RNA integrity was assessed using an Agilent 2100 Bioanalyzer to ensure high-quality RNA (RNA integrity number, RIN > 7.0) before proceeding with library preparation. For RNA sequencing library construction, RNA was sheared and purified using the TruSeq RNA Sample Preparation Kit v2 (Illumina), followed by reverse transcription into double-stranded cDNA (ds-cDNA). The cDNA was purified using the QIAquick Gel Extraction Kit (QIAGEN) and AMPure XP beads (Beckman Coulter) to obtain DNA fragments of 250–300 bp in length. DNA concentrations were quantified using the dsDNA HS Assay Kit on a Qubit 2.0 Fluorometer (Thermo Fisher Scientific), ensuring a minimum concentration of 1 ng/μL. Sequencing libraries were prepared and subjected to paired-end sequencing on an Illumina NextSeq 500 platform (Mid Output Kit v2) following standard sequencing protocols.

To analyze TSC marker expression, raw sequencing reads underwent quality control and preprocessing. Illumina sequencing adapters were trimmed using BBDuk (BBMap v37.87) with parameters specified in the Lexogen data analysis protocol. High-quality reads were aligned to the human reference genome (GRCh38/hg38) using RSEM with the Bowtie2 aligner. Gene expression levels were normalized using EDASeq and read counts per gene were calculated using SAMtools (v1.3.1) and HTSeq-count (v0.6.0). Genes with a mean count of less than 20 across all samples were removed to reduce noise. Differentially expressed genes (DEGs) were identified using edgeR, applying the following significance thresholds: *p* < 0.01, log fold change (LogFC) > 0.1, and an expression difference (pct1—pct2) > 0.05. Spearman’s correlation analysis was performed to examine overall gene expression patterns.

To investigate the biological significance of the identified DEGs, Gene Ontology (GO) term analysis and Kyoto Encyclopedia of Genes and Genomes (KEGG) pathway enrichment analysis were conducted using the ClusterProfiler package (version 3.6.0, R Bioconductor). Heatmaps were generated using the pheatmap package (version 1.0.10, R Bioconductor) to illustrate gene expression patterns, while volcano plots were created using ggplot2 (version 2.2.1, R Bioconductor) to visualize significantly differentially expressed genes. All computational analyses were performed in R (version 4.1.0) and Python (version 3.8).

### Assay for transposase-accessible chromatin with high-throughput sequencing (ATAC-Seq) analysis

ATAC-Seq was performed to assess chromatin accessibility following the protocol provided by the ATAC-Seq Assay Library Construction Kit (Vazyme, Cat# TD502-02). Cells were harvested and processed according to the manufacturer’s instructions. Transposase-mediated tagmentation was conducted to fragment open chromatin regions, and DNA libraries were prepared using the TruePrep DNA Library Preparation Kit V2 for Illumina (Vazyme, Cat# TD503-01). Constructed ATAC-Seq libraries were quantified using a Qubit dsDNA HS Assay Kit (Thermo Fisher Scientific, Q32851) and validated for appropriate fragment size distribution using an Agilent 2100 Bioanalyzer (Agilent Technologies, CA, USA). High-quality libraries were sequenced on an Illumina NextSeq 500 platform, generating paired-end 75-bp reads.

Raw sequencing reads underwent quality control (QC) filtering using FastQC (v0.11.9) to assess read quality, adapter contamination, and duplication rates. High-quality reads were trimmed using Cutadapt (v1.13) to remove adapter sequences and low-quality bases. For genome alignment, trimmed reads were mapped to the human reference genome (UCSC hg38) using Bowtie2 (v2.2.5) with default parameters optimized for ATAC-Seq analysis. Mapped reads were further processed by SAMtools (v1.3.1) for sorting and indexing, followed by de-duplication using Picard Tools (v1.90) to remove PCR duplicates and ensure data accuracy. To identify open chromatin regions, peak calling was performed using MACS2 (v2.2.7.1) with the callpeak function, and the bdgcmp function was applied to remove duplicate reads and improve peak specificity. Differential peak analysis between conditions was conducted using MACS2 bdgdiff, allowing for the identification of chromatin regions with significant accessibility differences.

Signal intensity heatmaps and read density profiles were generated using DeepTools (v2.4.2) to visualize global chromatin accessibility patterns. Motif enrichment analysis was performed using HOMER (v4.11) to identify transcription factor binding motifs enriched in open chromatin regions. To investigate the biological significance of differential chromatin accessibility, Gene Ontology (GO) term enrichment and Kyoto Encyclopedia of Genes and Genomes (KEGG) pathway analysis were performed using ClusterProfiler (v3.6.0). Statistical significance was determined using Benjamini–Hochberg multiple testing correction, with a false discovery rate (FDR) < 0.05 considered significant. All computational analyses were conducted using R (v4.1.0) and Python (v3.8) to ensure reproducibility and robustness in data interpretation.

### Statistical analysis

For all experiments, at least three independent biological replicates were performed to ensure reproducibility and statistical robustness. Statistical analyses were conducted using SPSS 22.0 (IBM, Armonk, NY, USA) and GraphPad Prism 9.0 (GraphPad Software, San Diego, CA, USA). Data are presented as mean ± standard error of the mean (SEM) unless otherwise specified. For comparisons between two groups, statistical significance was assessed using an unpaired two-tailed Student’s t-test. For multiple group comparisons, one-way analysis of variance (ANOVA) followed by Tukey’s post hoc test was applied to determine significant differences. All statistical tests were performed under parametric assumptions, and data distribution normality was verified using the Shapiro–Wilk test. For non-normally distributed data, appropriate non-parametric tests, such as the Mann–Whitney U test for two-group comparisons and the Kruskal–Wallis test for multiple groups, were applied. A *p*-value < 0.05 was considered statistically significant, with significance levels indicated as follows: (*) *p* < *0.05*, (**) *p* < *0.01*, and (***) *p* < *0.001*.

## Supplementary Information

Supplementary Material 1. Fig. S1: PD0325901 enhances the induction of GATA3^+^ cells. Fig. S2: Derivation of hTSCs from UH10 hiPSCs using YP medium. Fig. S3: Impact of WNT signaling on the derivation of hTSCs using YP medium.

Supplementary Material 2. Table 1: List of primers for RT-qPCR assay.

## Data Availability

The datasets (GSE118808, GSE144994, GSE166401, GSE192917, GSE135696) analyzed in this study were available from the Gene Expression Omnibus (GEO) database (https://www.ncbi.nlm.nih.gov/geo/query/acc.cgi). The RNA-seq and ATAC-seq data have been deposited in the GEO database under the accession code GSE306221 (Enter token cvkpqkeynjqrfgj into the box).

## Abbreviations

AME: Amniotic ectoderm
ANOVA: One-way analysis of variance
ATAC-Seq: Assay for transposase-accessible chromatin with high-throughput sequencing
cDNA: Complementary DNA
CGB: Chorionic gonadotropin beta subunit
DEGs: Differentially expressed genes
EdU: 5-Ethynyl-2'-deoxyuridine
EGF: Epidermal growth factor
EGFR: Epidermal growth factor receptor
ELISA: Enzyme-linked immunosorbent assay
ELF5: E74 like ETS transcription factor 5
EPCAM: Epithelial cell adhesion molecule
EPI: Epiblast
ERK: Extracellular signal-regulated kinase
EVT: Extravillous trophoblast
FDR: False discovery rate
FGF: Fibroblast growth factor
FOXA2: Forkhead box A2
GATA3: GATA binding protein 3
GO: Gene Ontology
hCG: Human chorionic gonadotropin
HLA-G: Human leukocyte antigen G
hPSCs: Primed human pluripotent stem cells
hTSCs: Human trophoblast stem cells
KEGG: Kyoto Encyclopedia of Genes and Genomes
KRT7: Keratin 7
MEK: Mitogen-activated protein kinase
MYB1: MYB transcription factor 1
NANOG: Nanog homeobox
OCT4: Octamer-binding transcription factor 4
PAX6: Paired box 6
PCA: Principal component analysis
PDGF: Platelet derived growth factor
RT-qPCR: Real-time quantitative polymerase chain reaction
SAVECY: SB431542, A83-01, valproic acid, epidermal growth factor, CHIR99021, Y-2763
SDC1: Syndecan 1
SEM: Standard error of the mean
SOX1: SRY-box transcription factor 1
SOX2: SRY-box transcription factor 2
SOX17: SRY-box transcription factor 17
STB: Syncytiotrophoblast
TE: Trophectoderm
TFAP2C: Transcription factor AP-2 gamma
TFAP2A: Transcription factor AP-2 alpha
TGF-β: Transforming growth factor-beta
TP63: Tumor protein p63
WNT: Wingless/Integrated
