# Supplementary material for: Transient inhibition of MEK/ERK and WNT pathways enhances direct differentiation of primed hPSCs into functional trophoblast stem cells
Source: Cell Regen. 2026 Jan 20;15:4. doi: 10.1186/s13619-025-00261-x (PMC12816472; doi:10.1186/s13619-025-00261-x)
Supplement: Supplementary file 1 — Supplementary Material 1. Fig. S1: PD0325901 enhances the induction of GATA3+ cells. Fig. S2: Derivation of hTSCs from UH10 hiPSCs using YP medium. Fig. S3: Impact of WNT signaling on the derivation of hTSCs using YP medium. [file 13619_2025_261_MOESM1_ESM.docx]

**Supplementary Fig. 1. PD0325901 enhances the induction of GATA3^+^ cells.** A. Cells were treated with YP medium for 1 day, 2 days, 3 days, and 4 days. Next, the YP medium was changed to TS medium, and cells were further cultured for 5 days and 10 days. Morphology of cells in different time points was recorded. Scale bar: 200 μm. B. RT-qPCR analysis of pluripotency (OCT4, SOX2), trophoblast (ELF5, GATA3, KRT7, TP63, TFAP2C), and epithelial (EPCAM) markers in YP-derived hTSCs. Error bars represent mean ± SD (n = 3). C. Immunostaining for OCT4 and GATA3 on Days 5 and 10 under TS, TPD, and YP conditions. Scale bar: 50 μm. D. Western blot analysis of MEK/ERK proteins under TS, TPD, and YP conditions. E. RT-qPCR of TSC markers (GATA3, KRT7, EGFR, TP63) under single-factor induction protocols. F. Cell cycle analysis of hTSCs across experimental groups. *P < 0.05, **P < 0.01, ***P < 0.001.


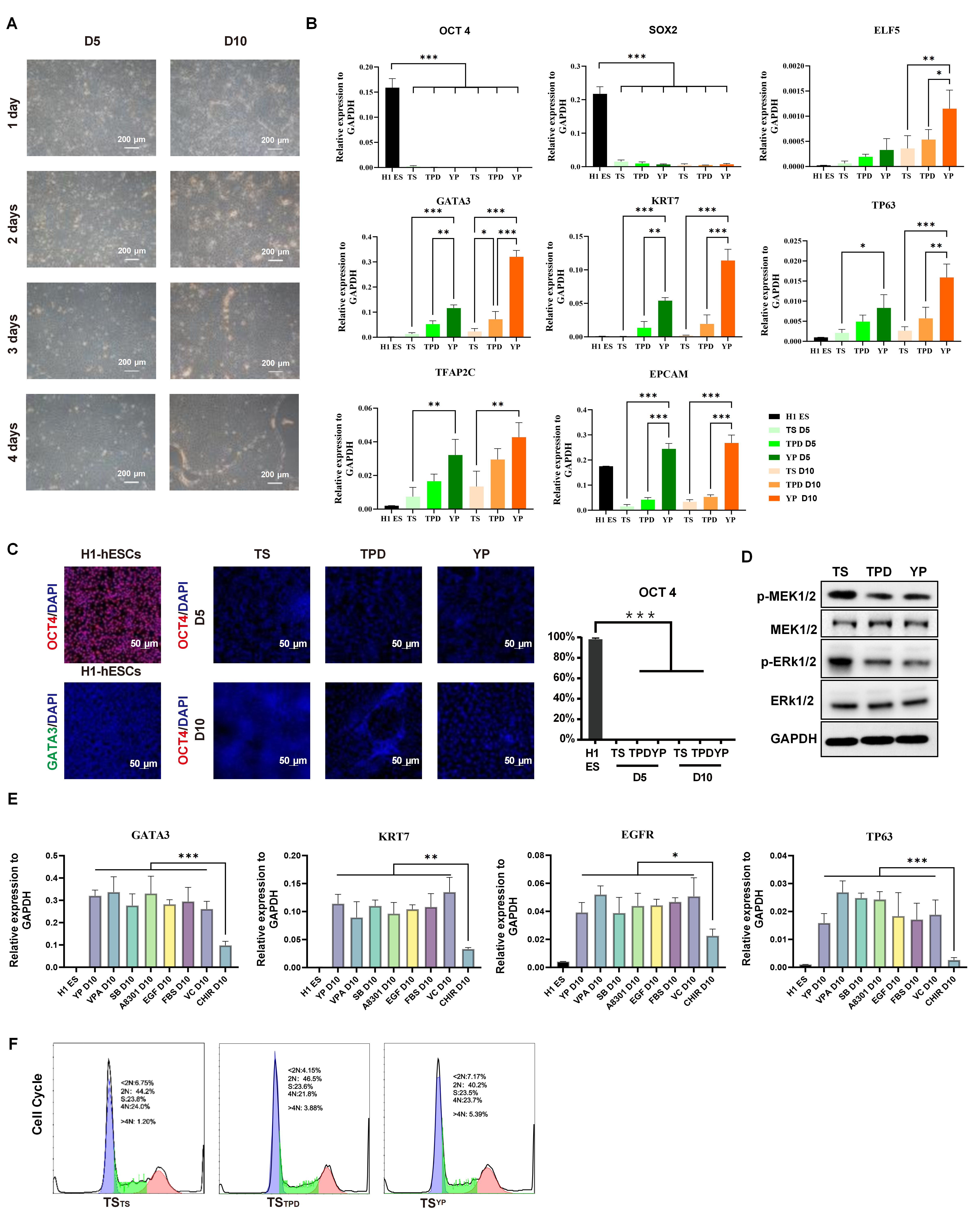


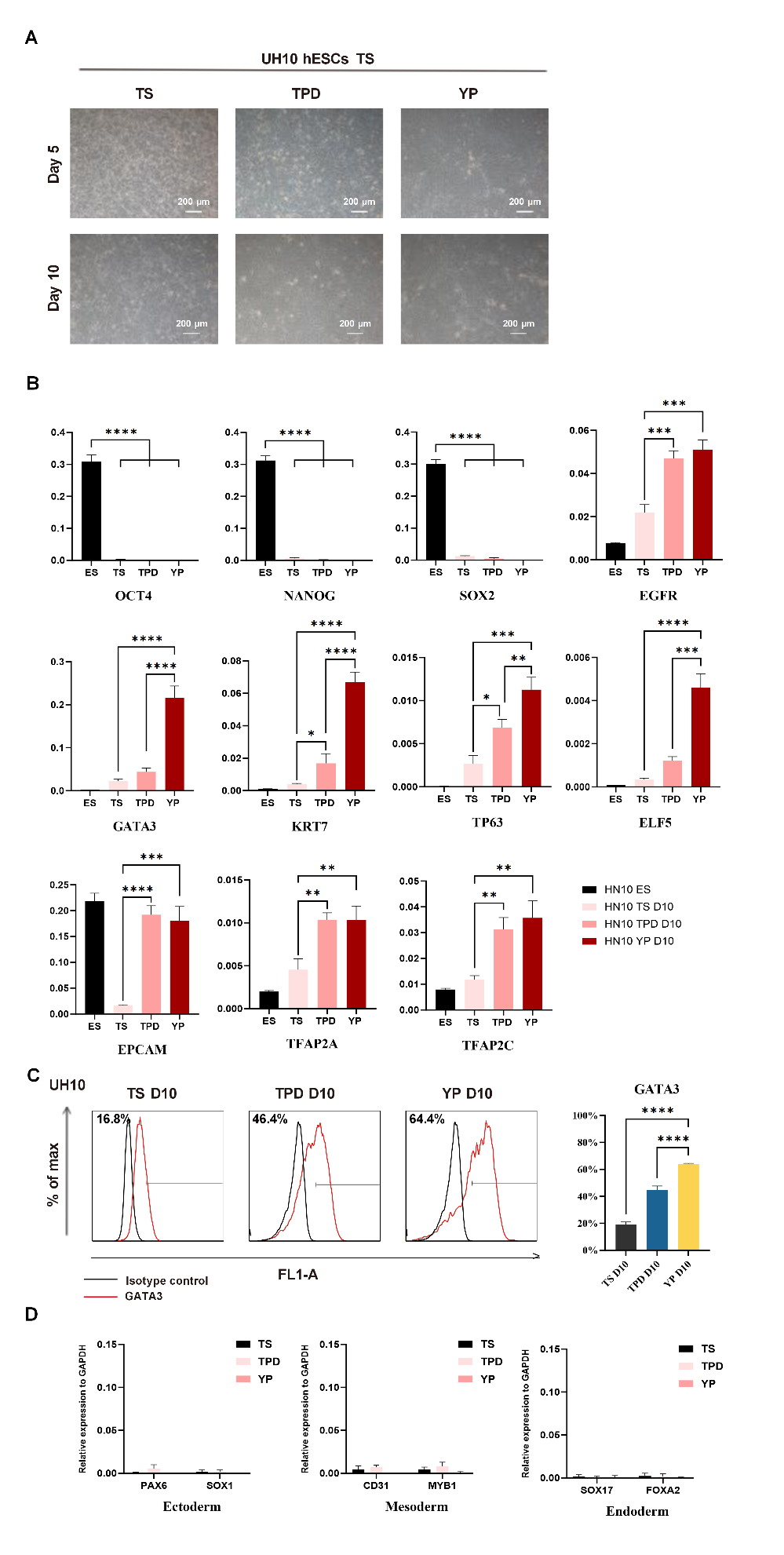


**Supplementary Fig. 2**. **Derivation of hTSCs from UH10 hiPSCs using YP medium.** A. Morphology of UH10 hiPSC-derived cells on Days 5 and 10 under TS, TPD, and YP conditions. Scale bar: 200 μm. B. RT-qPCR analysis of pluripotency (OCT4, NANOG, SOX2), trophoblast (ELF5, GATA3, KRT7, TP63, TFAP2A, TFAP2C, EGFR), and epithelial (EPCAM) markers in hiPSC-derived hTSCs. Error bars: mean ± SD from three replicates. C. Flow cytometry of GATA3⁺ cells in UH10-derived hTSCs under each condition. D. RT-qPCR analysis of expression of marker genes for the three germ layers, including PAX6/SOX1 (ectoderm), CD31/MYB1 (mesoderm), and SOX17/FOXA2 (endoderm) under TS, TPD, and YP conditions. *P < 0.05, **P < 0.01, ***P < 0.001.


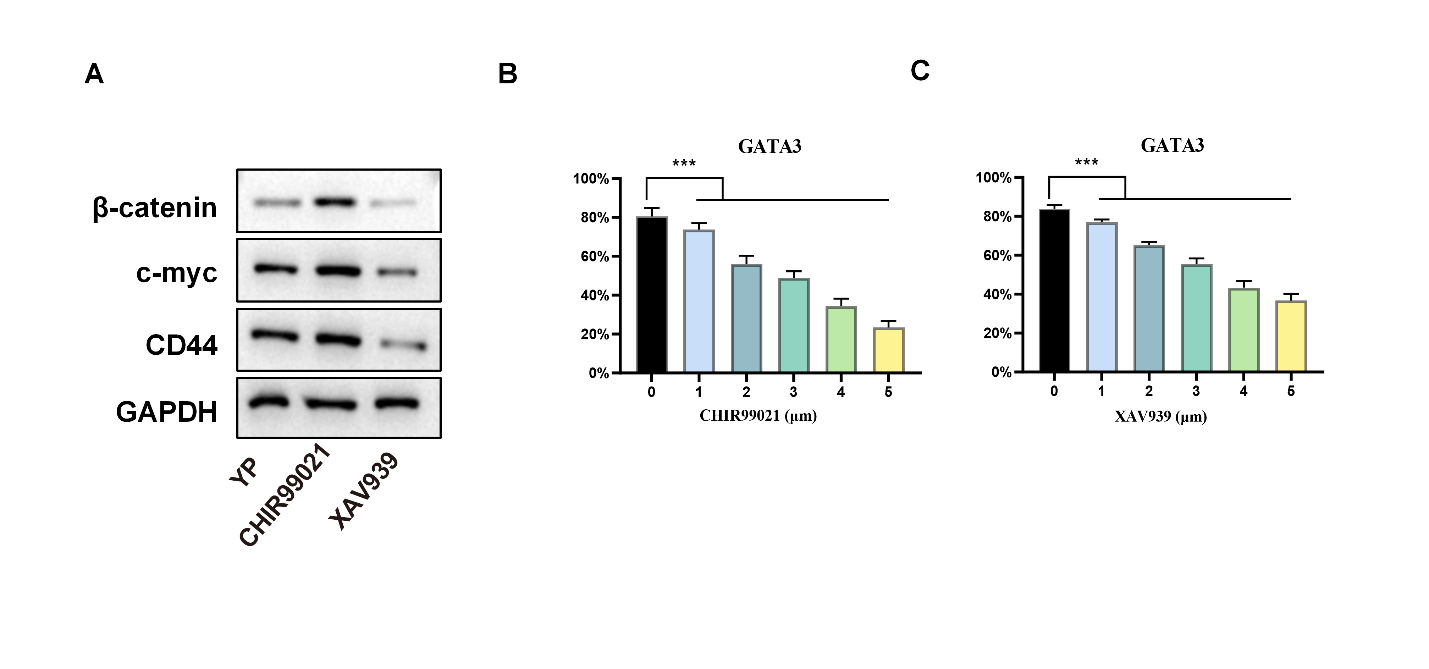


**Supplementary Fig. 3**. **Impact of WNT signaling on the derivation of hTSCs using YP medium.** A. Western blot analysis validated the activation and inhibition of WNT signaling under activator (CHIR99021) or inhibitor (XAV939) treatment. B-C. Flow cytometry analysis of GATA3⁺ cells under treatment with different concentrations of CHIR99021 or XAV939 in YP medium. ***P < 0.001.
