# Supplementary material for: Transient inhibition of MEK/ERK and WNT pathways enhances direct differentiation of primed hPSCs into functional trophoblast stem cells
Source: Cell Regen. 2026 Jan 20;15:4. doi: 10.1186/s13619-025-00261-x (PMC12816472; doi:10.1186/s13619-025-00261-x)
Supplement: Supplementary file 2 — Supplementary Material 2. Table 1: List of primers for RT-qPCR assay. [file 13619_2025_261_MOESM2_ESM.docx]

**Supplementary Table 1. List of primers for RT-qPCR assay.**

| **Gene** | **Forward (5’-3’)** | **Reverse (5’-3’)** |
| --- | --- | --- |
| GATA3 | GCTTCACAATATTAACAGACCC | TTAAACGAGCTGTTCTTGGG |
| KRT7 | TGTGGATGCTGCCTACATGAGC | CAATCTCCTGCTTGGTGTTGCG |
| TP63 | GCCCCTCCTAGTCATTTGAT | ATCCCTCCAACACAACTGCT |
| OCT4 | GAGAACCGAGTGAGAGGCAACC | CATAGTCGCTGCTTGATCGCTTG |
| SOX2 | GCCGAGTGGAAACTTTTGTCG | GGCAGCGTGTACTTATCCTTCT |
| ELF5 | GATCTGTTCAGCAATGAAG | GGTCTCTTCAGCATCATTG |
| TFAP2C | TCAGTCCCTGGAAGATTGTCG | CCAGTAACGAGGCATTTAAGCA |
| EPCAM | CTCCACGTGCTGGTGTGT | TGTTTTAGTTCAATGATGATCCAGTA |
| EGFR | AACTGTGAGGTGGTCCTTGG | AACTGTGAGGTGGTCCTTGG |
| NANOG | CTCCAACATCCTGAACCTCAGC | CGTCACACCATTGCTATTCTTCG |
| PAX6 | TGGAGTGTACTGAGGAATCTG | AAGGAGTTGCTGGTGAGAG |
| SOX1 | CACCCGGATTACAAGTACC | TACTTGTCCTTCTTGAGCAG |
| CD31 | GAGAAGAAAGAATCCTTCTCTACAC | TAATGTGGAGCTGAGCTCC |
| MYB1 | AATGTCTCCAGTCATGTTCC | TATAGTGTCTCTGAATGGCTG |
| SOX17 | TGAACGCTTTCATGGTGTG | TGTGCAGGTCTGGATTCTG |
| FOXA2 | CGAGTTAAAGTATGCTGGGAG | GTTCATGTTGCTCACGGAG |
| CGB | GTCAACACCACCATCTGTG | TAGTTGCACACCACCTGAG |
| GCM1 | TGAAGAAAGTGAACACAGCA | TTTCACCTGGAAGAGACCT |
| ENDOU | GTGTAGTGACCACGAGGTC | CTGTAGATCTTCTCAGAGATGCT |
| HLA-G | TGAGATGGAAGCAGTCTTC | AGTGACTACAGCTGCAAGG |
| GAPDH | GTCTCCTCTGACTTCAACAGCG | ACCACCCTGTTGCTGTAGCCAA |
